# Current concepts on feeding the critically ill patient: a narrative review

**DOI:** 10.1186/s13054-026-06123-5

**Published:** 2026-06-12

**Authors:** Michael Adolph, Arthur R. H. van Zanten, Mette M. Berger, Sirak Petros, Michael Hiesmayr, Arved Weimann, Marc E. Martignoni, Katharina Feil, Simon Hirschberger, Valerie Haller, Carla Wunderle

**Affiliations:** 1 Department of Anesthesiology and Intensive Care Medicine and Department of Nutrition Management, University Clinic Tübingen, 72074 Tübingen, Germany; 2 Department of Intensive Care, Gelderse Vallei Hospital, Willy Brandtlaan 10, 6716 RP Ede, The Netherlands; 3 Division of Human Nutrition and Health, Wageningen University & Research, Helix (Building 124), Stippenweg 4, 6708 WE Wageningen, the Netherlands; 4 Faculty of Biology and Medicine, Lausanne University, 1015 Lausanne, Switzerland; 5 Medical ICU, University of Leipzig Medical Centre, 04103 Leipzig, Germany; 6 Center for Medical Data Science, Institute for Medical Statistics, Medical University Vienna, 1090 Vienna, Austria; 7 Department of General, Visceral and Oncological Surgery, St. George Hospital, 04129 Leipzig, Germany; 8 Department of Surgery, School of Medicine, TUM University Hospital, Technical University of Munich, 81675 Munich, Germany; 9 Department of Neurology, University of Ulm (UKU), 89081 Ulm, Germany; 10 Department of Anaesthesiology, LMU University Hospital Munich, 80336 Munich, Germany; 11 Walter-Brendel-Centre of Experimental Medicine, Ludwig Maximilians University, 80539 Munich, Germany; 12 Medical University Department, Division of General Internal Medicine, Endocrinology and Family Medicine, Kantonsspital Aarau, Tellstrasse 25, 5001 Aarau, Switzerland; 13 Department of Nutritional Science, Justus Liebig University, 35392 Giessen, Germany

**Keywords:** Nutrition therapy, Intensive care unit, Malnutrition, Individualised therapy, Outcomes, Nutritional targets, Ketogenic diet

## Abstract

**Background:**

Nutritional therapy is a key component of critical care management, yet optimal strategies remain debated due to the heterogeneity of ICU patients, dynamic metabolic alterations, and the profound influence of inflammation on nutrient utilisation. Evidence from recent trials has challenged traditional one-size-fits-all approaches, emphasising the need for individualised, phase-specific nutrition throughout the continuum of critical illness and recovery. This manuscript summarises current concepts and emerging evidence in nutrition therapy presented at the 39th Annual Conference of the German Society for Nutritional Medicine (DGEM). Experts reviewed and critically discussed inflammation-driven metabolic changes, personalised energy and protein prescriptions, micronutrient management, macronutrient adaptation, ketogenic strategies in neurocritical care and sepsis, and nutritional considerations in post-ICU syndrome and outpatient recovery. This overview does not claim to be exhaustive; interpretations of individual study results partly reflect the views of the experts. Together with the inclusion of newer therapeutic approaches, this is intended to stimulate discussion and, at the same time, provide a basis for further studies.

**Main body:**

Inflammation and high disease severity strongly influence nutritional responsiveness, with highly inflamed patients demonstrating reduced benefit and heightened risk of overfeeding. Personalised strategies, including indirect calorimetry, fat-free mass–based protein dosing, and metabolic biomarkers such as the urea-creatinine ratio, offer a rational framework for tailoring therapy. Micronutrient deficiencies are common due to redistribution, pre-existing deficits, and extracorporeal losses, necessitating structured assessment and supplementation. Macronutrient delivery should be progressively escalated and regarded as a pharmacologic intervention aligned with disease phase and organ function. Early standardised ketogenic diet protocols show feasibility and potential clinical benefit in refractory status epilepticus and sepsis. Post-ICU and outpatient phases remain nutritionally vulnerable, with persistent catabolism and underfeeding common; structured, multidisciplinary rehabilitation and transitional nutrition programs may improve long-term outcomes.

**Conclusion:**

Future personalised nutrition strategies may rely on metabolic phenotyping and biomarker-informed stratification rather than uniform protein, energy and micronutrient targets for all ICU patients. Integrating individualised energy and protein prescription, targeted micronutrient management, emerging metabolic therapies, and coordinated post-ICU rehabilitation may optimise recovery and functional outcomes. Robust clinical trials are needed to confirm the impact of these personalised strategies on long-term patient-centred endpoints.

## Background

**Fig. 1:**
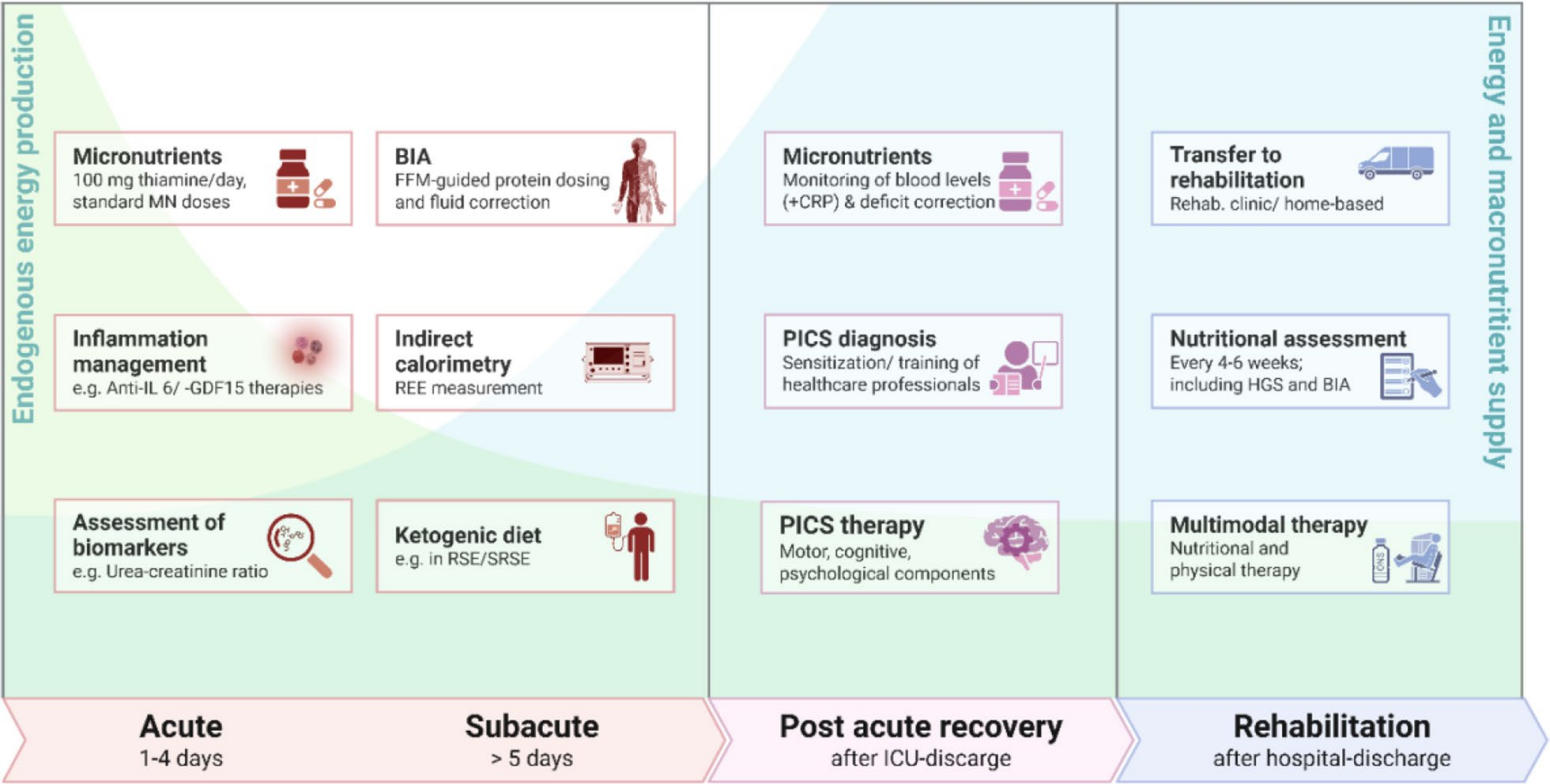
Phase-specific nutrition strategies across the continuum of critical illness and recovery. The figure illustrates key nutritional priorities during the acute, subacute, post-ICU recovery, and rehabilitation phases. Early care focuses on inflammation management, metabolic phenotyping, and cautious delivery of energy and micronutrients, recognising limited tolerance for exogenous substrates. During the subacute phase, nutritional strategies evolve with improving metabolic capacity, including progressive protein provision and targeted interventions. Post-ICU recovery emphasises diagnosis and management of post-intensive care syndrome (PICS), nutritional assessment, and continuity of care, while the rehabilitation phase integrates multimodal therapy combining nutrition, physical training, and long-term functional recovery. REE, Resting energy expenditure; IL6, Interleukin 6; GDF15, Growth differentiation factor 15; FFM, Fat free mass; RSE, Refractory status epilepticus; SRSE, Super-refractory status epilepticus; PICS, Post-intensive care syndrome; CRP, C-reactive protein; HGS, handgrip strength; BIA, Bioelectrical impedance analysis; ICU, intensive care unit

The optimal nutritional strategy for critically ill patients remains the subject of ongoing debate. Although large, randomised studies have been conducted comparing different nutritional strategies [1], none of them has demonstrated the superiority of a particular strategy for all patients. The focus was long placed on optimal energy delivery, and recently the focus has balanced in the direction of protein supply. More recent trials of higher protein provision to combat protein catabolism [2, 3] consistently demonstrated no benefit In the PRECISe study, higher doses of enteral protein led to a decline in health-related quality of life (primary outcome) among critically ill patients, whereas in the EFFORT Protein study, higher protein intake did not improve time to hospital discharge and, at the same time, outcomes worsened among patients with acute kidney injury and high organ failure scores. Isolating the impact of nutrition on outcomes is difficult, as heterogeneity increases and the number of comorbidities rises among adult Intensive Care Unit (ICU) patients [4]. Moreover, the substantial heterogeneity of ICU patients may limit the generalizability of study results, which should instead serve as a foundation for individualised clinical decision-making [5]. Thus, nutritional management in the acute phase of an illness remains a challenge (Fig. 1). This report summarises the current concepts of nutritional therapy in the ICU that were presented at the 39th scientific conference of the German Society for Nutritional Medicine (DGEM) from October 24 to 25, 2025, in Irsee, Germany.

### Current concepts of nutritional care during the patient journey of the critically ill patient

#### Inflammation – do we have to take individualised nutrition into account?

**Fig. 2:**
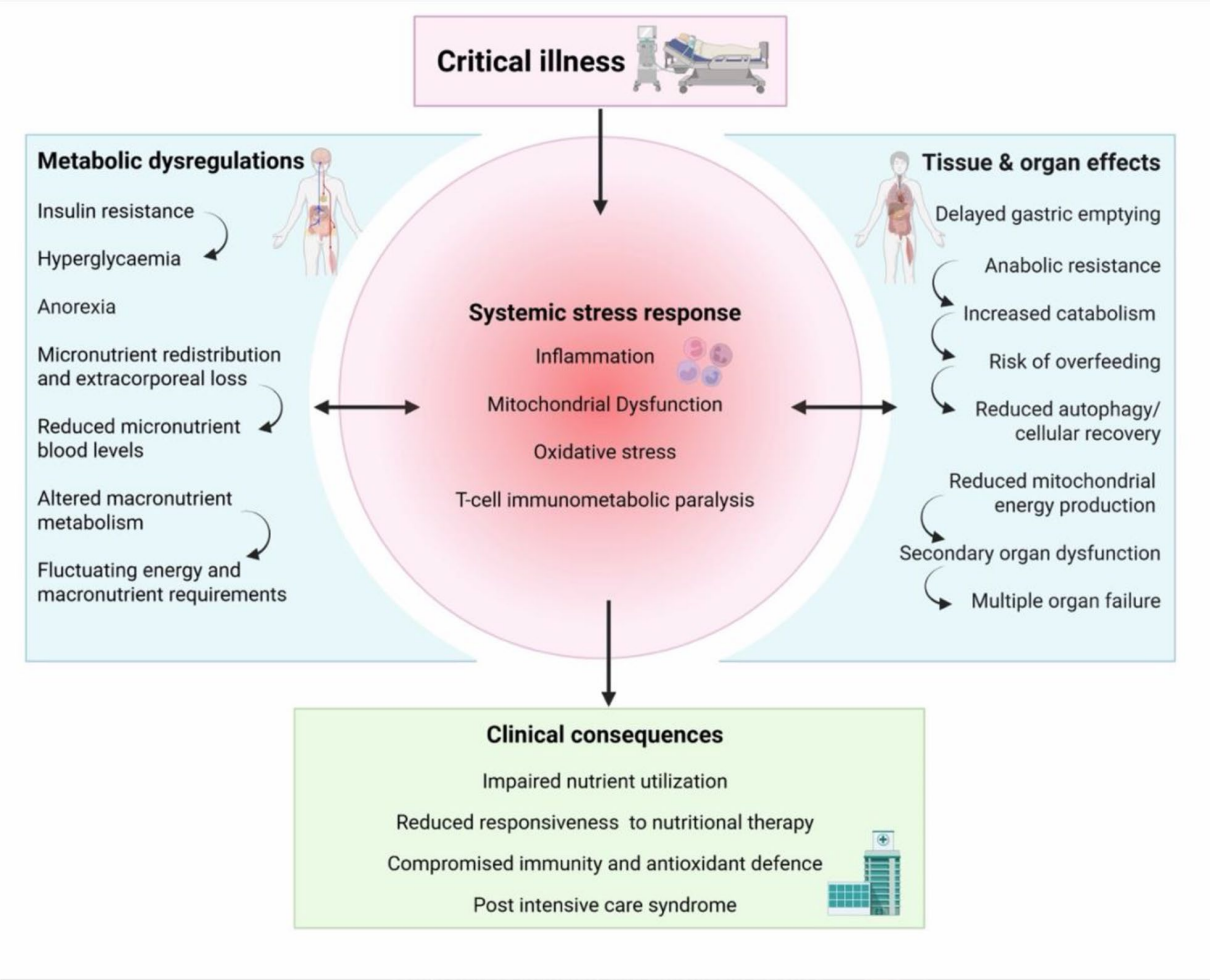
Systemic stress response as the central driver of metabolic dysfunction and impaired nutrition response in critical illness. Critical illness triggers a systemic stress response characterised by inflammation, mitochondrial dysfunction, oxidative stress, and immunometabolic dysregulation. This response underlies widespread metabolic disturbances, including insulin resistance, altered micronutrient distribution, anorexia, and fluctuating energy and macronutrient requirements. At the tissue and organ level, these processes contribute to anabolic resistance, impaired gastrointestinal function, reduced cellular energy production, and secondary organ dysfunction. Collectively, these mechanisms lead to impaired nutrient utilisation, reduced responsiveness to nutritional therapy, and adverse clinical consequences, including post-intensive care syndrome (PICS). PICS, post-intensive care syndrome

Inflammation is the natural response to certain triggers and involves complex, multi-stage biological processes involving metabolic, endocrine, and immunological pathways (Fig. 2) [6, 7]. However, problems can arise when the inflammatory response persists or is excessive [8]. In addition, the influence of inflammation on our metabolism has long been underestimated and is so significant that it can shape nutritional needs and responses across diverse clinical phenotypes [9].

Inflammation is a major driver of malnutrition and appears to influence responsiveness to nutritional therapy, which has only been shown in non-critically ill patients [10, 11]. A sub-analysis of the EFFORT cohort demonstrated that patients with excessive inflammation (C-reactive protein [CRP] > 100 mg/dL or Interleukin-6 [IL-6] > 11.17 pg/mL) did not show the same survival benefit from nutrition therapy as patients with lower inflammation [11, 12]. Mechanistically, highly inflamed patients exhibit, among other things, disease-related anorexia, delayed gastric emptying, hyperglycemia with insulin resistance, and extensive endogenous tissue breakdown, all of which impair effective nutritional utilisation [9, 13]. Their increased catabolism raises the risk of overfeeding, but currently there are no practical tools for everyday use to measure or accurately estimate catabolism [14]. Routine biomarkers, glucose, albumin, urea, triglycerides, CRP, and potentially Growth/differentiation factor 15 (GDF15), offer partial insight but cannot fully capture inflammatory complexity. Overfeeding may also impair autophagy, potentially hindering cellular recovery [15]. New therapeutic approaches include the targeted manipulation of the inflammatory cascade. This could be a way to enable nutritional therapy in the acute phase of a disease. Promising but still exploratory are approaches such as blocking GDF15 or using anti-IL6 therapies [16, 17]. In a study with Ponsegromab (GDF-15 antibody), an improvement in functionality and stabilization of body weight was already observed in patients with cachexia [16].

In acutely and severely inflamed patients, full nutritional therapy faces multiple physiological barriers, underscoring the need for individualised, phase-specific, and gradually intensified nutritional strategies.

#### Personalised nutrition therapy in critical care: how to monitor and control it successfully?

Critical illness induces profound metabolic alterations characterised by marked catabolism, anabolic resistance, and rapid skeletal muscle wasting. Puthucheary et al. show that the rectus femoris decreased by 12.5% within the first 7 days [18]. Traditional “one-size-fits-all” strategies for energy and protein prescription are inadequate because resting energy expenditure (REE) and fat-free body mass (FFM) vary widely between patients and change dynamically over time. Predictive energy equations are highly inaccurate, with potentially large individual deviations; therefore, their clinical use in the acute phase should be reconsidered [19, 20].

A personalised approach begins with indirect calorimetry to measure REE, as recommended in the adult ICU nutrition guidelines by the European Society for Clinical Nutrition and Metabolism (ESPEN). The first step to nutrition individualisation is to achieve a body weight adapted target at day 5–7 progressively to account for the substantial, non-inhibitable endogenous energy production characteristic of early critical illness [14, 21–23]. At this point, indirect calorimetry may help to further individualise treatment.

Current pathophysiological considerations suggest that protein dosing may be guided by FFM rather than total body weight. Bioelectrical impedance analysis (BIA) allows estimation of FFM and correction for fluid overload in a stable phase [24]. However, the superiority of this approach has not yet been demonstrated, as no studies have been conducted to date to compare the two methods. Taken together, recent trials support caution with early high-dose protein provision and suggest that moderate protein doses, approximately 1.0–1.2 g/kg/day in many ICU populations, may be a pragmatic and safer target until more precise phenotype-guided strategies have been validated [2, 3, 25]. High protein doses early in the ICU course should be avoided, with gradual advancement towards the target over the first 4–5 days [21, 26].

In this context, attention has increasingly shifted towards metabolic markers that may help explain why some critically ill patients appear less tolerant of early high protein exposure. A high urea-to-creatinine ratio should not yet be considered a validated endotype-defining biomarker but may represent a candidate marker of a metabolically distinct ICU subgroup characterised by increased protein catabolism, enhanced ureagenesis and potentially reduced efficiency of exogenous protein utilisation [27]. Increased urea cycle activity appears to mediate the mortality signal observed in high-protein groups in randomised trials, suggesting that intolerance to high protein intake, rather than protein itself, may drive harm [28].

Future personalised nutrition strategies in the ICU may integrate indirect calorimetry–guided energy dosing, protein prescription based on fat-free mass, progressive advancement of nutrition during the first days of critical illness, and the incorporation of metabolic biomarkers such as the urea-creatinine ratio. This precision approach has strong mechanistic rationale and the potential to optimize outcomes across the ICU trajectory, although robust evidence demonstrating improved clinical endpoints is still limited.

#### Micronutrients and their importance in individualised nutrition for critically ill patients

The acute phase of critical illness is characterised by intense oxidative stress, mitochondrial dysfunction, and the rapid development of inflammation, that contribute to secondary organ dysfunction and multiple organ failure. Micronutrients (MNs), i.e. trace elements and vitamins, are essential constituents of endogenous antioxidant defences and immune defences. Mitochondrial energy production, which is regulated by the B vitamin family, is acutely compromised, an alteration which may persist and contribute to organ failure [29].

The presence of inflammation increases the oxidative stress, and causes redistribution of the MNs across organs, decreasing blood levels [30]. This decrease is proportional to the intensity of the inflammatory response: only copper increases [30]. Simultaneous determination of CRP is required to assess the MN status [31].

Critically ill patients may have MN status alterations before admission due to disease and further they require treatments like renal replacement or extracorporeal membrane oxygenation (ECMO), which although lifesaving, cause MN losses, further compromising MN related immunity and antioxidant defense. Some MNs are critical [31]: Thiamin B1 (glucose metabolism and energy production), Niacin B3 (NAD synthesis and redox reactions), ascorbic acid (major circulating antioxidant), Vitamin D (immunity, multiple other actions), iron (oxygen transport, energy production, immunity), selenium (antioxidant defense, immunity), and zinc (antioxidant defense, wound healing, immunity). The n-3 polyunsaturated fatty acids (PUFA) eicosapentaenoic acid (EPA) and docosahexaenoic acid (DHA), although not MNs, are essential for the resolution of inflammation, and muscle maintenance) [32].

Administration from admission of thiamine 100 mg/d and standard doses for parenteral nutrition doses of MNs for about 5 days completes the insufficient intakes due to associated with the progressive nutrition strategy, where feeding dose is reached only about 5 days after its initiation (MN delivery is then below daily recommended intake): no analytical outwork is required for such prescription. Prior MN deficits require specific treatment based on history and blood determination for diagnosis. Monitoring of blood levels, always with CRP, enables prescribing and correcting deficiencies and should be done in patients requiring medical nutrition therapy beyond one week, especially in those with biological fluid losses (continuous renal replacement therapy, fistulae, exudates, etc.) that require repletion to restore a normal status. While preventing and treating deficits has proven benefits, high-dose single MN administration should not be done [31].

#### Macronutrients – when, which and how within the scope of personalised nutrition therapy

In addition to micronutrients, determining the optimal macronutrient supply for critically ill patients remains a challenge. Due to the large heterogeneity of the critically ill patient population, a one-size-fits-all strategy cannot be a solution [20]. Nutrition guidelines define the energy metabolism of the critically ill into ebb and flow phases based on the findings of Cuthbertson in trauma patients [21, 33]. However, the extent of the acute clinical condition, chronic comorbidities, the increasing proportion of elderly patients and clinical frailty contribute to a considerable heterogeneity of the critically ill. A considerable proportion of the critically ill also undergoes a variable clinical course during the ICU stay either due to the admission diagnosis or complications [34].

Although measuring energy expenditure with indirect calorimetry helps further understand the metabolic state, it is not sufficient to define the patient’s macronutrient needs. Endogenous glucose production following metabolic adaptation plays a significant role, but unfortunately, cannot be quantified during routine patient care [14, 35]. The guideline recommendations for protein supply also represent pragmatic suggestions due to the lack of well-conducted clinical trials at the time of guideline development [21]. The focus on protein metabolism remains on its impact on skeletal muscle breakdown and repair [36]. However, inflammation following trauma, infection or non-infectious conditions results in the synthesis of a myriad of mediators for which amino acids are required. Thus, the extent of inflammation has a significant impact on macronutrient metabolism [11]. Protein metabolism is also modified by acute kidney injury and kidney replacement therapy. Amino acid infusion in a selected patient population may have a prognostic impact beyond nutrition therapy [37]. Therefore, macronutrient supply during critical illness should be considered as a pharmacotherapy and be personalised accordingly. There is yet a challenge in identifying sensitive and specific biomarkers to define the daily macronutrient requirements of the critically ill.

#### The critically ill: rational nutrition individualisation

The aim of nutrition care in the critically ill is to provide sufficient macronutrients, such as glucose, lipids, and protein/amino acids, for energy metabolism as well as the maintenance and repair of tissues. Energy metabolism is dependent on a continuous supply of nutrients and oxygen to produce ATP in cells. ATP stored in cells is only sufficient to maintain the activity of energy-consuming processes for about 1–2 min. Insufficient ATP production is immediately associated with loss of function. Glucose is a critical nutrient in some tissues, such as the brain, which has no relevant glycogen stores; thus, the human body reacts to injury with a strong stimulation of gluconeogenesis that persists for 10 days in the ICU [14]. Lipolysis is also strongly stimulated in early critical illness. Amino acids for maintenance and repair can be provided by intracellular protein breakdown, but this is associated with a loss of fat free mass (FFM). In summary, during the acute phase of critical illness, metabolic changes trigger an endogenous catabolic response. The body breaks down its own fat, glycogen, and muscle stores to maintain ATP production, regardless of external nutrition. This explains why energy levels remain stable despite a progressive increase in external feeding [22].

A sub analyses of the EPANIC trial [38] and a recent mechanistic study [39] demonstrate that administering higher protein/amino acid loads during the acute phase of critical illness often results in metabolic inefficiency. Instead of being utilised for muscle synthesis, the excess protein is broken down and converted into urea [28].

Early critical illness is characterized by provision of all nutrients necessary to maintain ATP production from breakdown of body stores and gluconeogenesis with amino-acids from protein breakdown. Lower ATP production as estimated from oxygen consumption despite sufficient nutrients and oxygen is associated with higher mortality [40, 41].

Unfortunately, when nutrients are provided via oral, enteral or parenteral nutrition there is no method to estimate the relative contribution of internal breakdown to the total amount of nutrients utilised for ATP and protein synthesis. In a retrospective study, ICU mortality was lowest among patients who received approximately 70% of measured energy consumption as oral, enteral or parenteral nutrition during the first week in critical illness [40]. While these findings have been interpreted as suggesting a potential contribution of endogenous energy production during the acute phase that may not be fully suppressible by exogenous nutrient provision, this hypothesis remains exploratory. These observations support the concept that full replacement of measured energy expenditure may not always be appropriate during the earliest phase of critical illness [21]. The German guidelines suggest adjusting energy intake based on insulin requirements and phosphate levels [42], a pragmatic approach that may need to be tested in an interventional study. Other approaches aim to develop further bedside methods to estimate transition from catabolism to anabolism using an insulin resistance index, potentially refining the timing and individualisation of nutritional therapy [43].

The most recent guidelines recommend a progressive increase in nutrient provision, aiming to reach the target around days 5–7 in the ICU. Guidelines [21, 42, 44] are heterogeneous regarding the starting amount, route and timepoint. Adaptation to obesity is mentioned but not to age or sex. Renal injury and replacement therapy may necessitate specific adaptations [45], less protein with reduced renal function and additional amino acids during renal replacement therapy to compensate for typical losses of 10–25 g of amino acids via the hemofilter.Observational and interventional studies have shown that there is substantial variability in the amount of nutrients provided [2, 46, 47]. In the nutritionDay cohort of 24,726 patients from 68 countries, we found that after 7 days in ICU [48] fewer than one third of patients achieved the recommended nutritional targets, while approximately one third were overnourished and another one third were undernourished. Protein provision is often lower than typically recommended, but large trials suggest that high-dose protein in the ICU is associated with an increased risk. Recent observations suggest that nitrogen excretion derived from urinary urea production and the urea/creatinine ratio may provide an easy way to assess protein supply [28, 49].

The first step in nutrition individualisation is to use a body weight adapted target. Progressively reaching the target at days 5–7 assumes that endogenous nutrient production may have decreased. This assumption may not apply in patients with an uncontrolled inflammatory process, phases of shock or surgical interventions [46, 50]. At this point, indirect calorimetry may help to further individualise treatment. The second step is to adapt protein supply based on urea production as a surrogate for protein breakdown, and to properly interpret the urea-to-creatinine ratio. We suggest that ordering nutrition as a rate rather than a 24-h dose may facilitate adaptation to patient characteristics and evolution. Since most enteral and parenteral nutrition has about 1 kcal/ml ordering a rate of 1 ml.kg^−1^.h^−1^ gives a target of 24 kcal.kg^−1^.day^−1^ and deviations are easily noticed.

Having outlined the general principles of clinical nutrition in the ICU, we now turn to a more specialised area that warrants brief consideration: the ketogenic diet. We recognise that this approach remains a niche intervention within critical care practice. Nevertheless, given the growing interest in its potential metabolic and therapeutic effects in select clinical contexts, we consider it relevant to include a concise overview here.

### Ketogenic diet in adult neurocritical care: from experimental concept to structured implementation

Refractory status epilepticus (RSE) and super-refractory status epilepticus (SRSE) remain among the most challenging neurological emergencies, with substantial morbidity and mortality [51–53]. Incidence estimates (~ 0.7/100,000) and the proportion of SE evolving to SRSE (5–10%) underscore the clinical burden [54, 55]. Evidence for late-line pharmacologic options is limited [56, 57], prompting in metabolic strategies such as the ketogenic diet (KD). Mechanistically, KD provides alternative energy source for neuronal metabolism and modulates excitatory–inhibitory balance, inflammation, and mitochondrial function [55, 58]. While this is well established in paediatrics and paediatric ICU, including new onset RSE (NORSE)/ Febrile Infection-Related Epilepsy Syndrome (FIRES), [59, 60] data from adult ICUs are heterogeneous and largely derived from non-randomised studies [61–66]. Preliminary phase I/II trials suggest that ketosis can be achieved within approximately two days and that seizures were stopped in most participants who completed the study, albeit with the expected metabolic side effects [62]. The implementation, however, is complicated by hidden carbohydrates and metabolic instability [54, 55]. To standardise care, we developed an interdisciplinary standard operating procedure (SOP) for classical 4:1 KD in adult ICU patients with RSE/SRSE, detailing indications, contraindications, initiation, and monitoring [67]. The SOP operationalises early initiation (when SRSE is anticipated), stepwise 4:1 escalation with optional medium-chain triglyceride (MCT) supplementation, daily metabolic checks with β-hydroxybutyrate (BHB) targeting 1–3 mmol/L, and predefined stop rules for severe acidosis or hypertriglyceridemia [67]. Importantly, comparative adult ICU data are now available: in a retrospective severity-matched cohort of critically ill adults with SRSE (18 KD vs. 16 controls) KD was feasible and safe. Time-dependent and multivariable Cox models suggested an association between KD, particularly earlier initiation, with increased likelihood of SRSE resolution, while unadjusted comparisons were confounded by delayed use and higher refractoriness [68]. Practically, KD can be delivered via nasogastric/percutaneous endoscopic gastrostomy tubes with point-of-care monitoring and requires rigorous pharmacy-led medication review to avoid ketosis-impairing carbohydrates. Taken together, KD is a plausible and implementable adjunct in adult RSE and SRSE when guided by a protocol and meticulous medication/nutrition review. Prospective multicentre evaluation should clarify efficacy, timing, and biomarker-guided patient selection.

### Ketogenic diet in critically ill patients

Systemic immune dysregulation is a hallmark of critical illness. While innate immunity elicits a profound inflammatory response to trauma or infection, adaptive immune cells, particularly T cells, enter a state of immunoparalysis, accompanied by severe metabolic defects [69, 70]. Recently, mitochondrial damage and dysfunction resulting in bioenergetic failure have been identified as the underlying cause of T-cell immunometabolic paralysis in critically ill patients [71]. Given the high impact of nutrition on cellular metabolism, nutritional interventions could offer a promising tool to restore mitochondrial function and immunometabolism in these patients.

Current protocols for enteral nutrition in intensive care units favour carbohydrate-rich formulations. Yet excessive carbohydrate intake may worsen immune dysfunction by triggering systemic inflammation through glucose-mediated activation of the NLRP3 inflammasome [72]. Limiting the fraction of carbohydrates to below 10% of total caloric intake induces hepatic ketogenesis, yielding ketone bodies—primarily BHB—as alternative energy sources [73]. This KD redirects human metabolism toward utilisation of fatty acids, thereby inducing an immunometabolic reprogramming that enhances mitochondrial oxidative phosphorylation and T-cell function [74, 75]. Clinical data on the KD in critically ill patients remain limited. McNelly et al. conducted a randomised pilot study in patients in critical care [76]. Plasma ketone bodies were significantly elevated in the KD group. BHB serum concentrations, however, only barely reached the threshold for stable ketosis. Nevertheless, the KD was well tolerated, and vasopressor requirements were significantly reduced in patients receiving the ketogenic feed. Rahmel et al. conducted a randomised controlled trial in critically ill adults with sepsis [77]. Here, patients receiving a KD achieved stable ketosis throughout the study period, again without adverse effects. The KD markedly improved glucose homeostasis, eliminated the need for insulin, and significantly increased ventilator-free, vasopressor-free, dialysis-free, and ICU-free days. In conclusion, these studies provide preliminary evidence that ketogenic nutrition is a feasible, safe, and metabolically effective intervention in critically ill patients, with potential clinical benefits. These findings support the rationale for larger trials to evaluate immunological effects and patient-centred outcomes.

### Post intensive care syndrome

During the past few years, the outcome of ICU survivors has drawn more attention and has led to the identification of Post-Intensive Care syndrome (PICS). The Society of Critical Care Medicine has defined PICS as “New or increased physical, cognitive or mental health impairments after ICU hospitalisation”. There is a consensus that PICS is a variable and complex syndrome that requires a holistic, individual, multidisciplinary, and multiprofessional approach including assessment and therapy of motor-, cognitive-, and psychological health impairments [78–83]. A recent multimodal rehabilitation guideline for patients with PICS has claimed that health care professionals must be sensitised and trained to recognise the condition [84]. Complex assessment of health functions will be mandatory. There is a need for early planning for a direct transfer to an interdisciplinary rehabilitation clinic or intensified home-based care.

Overall, nutrition must be part of the multimodal treatment bundle. Physical activity may enhance the benefits of nutritional therapy [21]. From a metabolic and nutritional standpoint, it is essential to ensure that a standardised swallowing assessment is performed to exclude dysphagia and aspiration risks before starting oral feeding [84]. Dysphagia interventions have shown significant impact on the incidence of pneumonia in a recent meta-analysis [85]. Additionally, diminished oral food intake may be due to loss of appetite, taste alteration, fatigue, cognitive impairment. Data indicate that only 50–70% of energy and protein requirements are met [86], which makes enteral complementation necessary [87]. These individuals require dedicated nutritional management and close monitoring. It is recommended to conduct nutritional assessments, including body composition analysis (BIA) and muscle function testing (handgrip strength), every 4–6 weeks for the first six months, followed by every three months thereafter [83]. Telerehabilitation may also help improve adherence to both physical exercise and nutritional interventions [88].

### Nutritional management in the outpatient setting

Patients transitioning from acute hospitalisation to community care experience profound and dynamic changes in nutritional status that strongly influence their long-term recovery trajectory. Although simplified, the metabolic and functional challenges can be divided into three clinical phases: acute illness, post-acute recovery, and home rehabilitation at home. A major challenge is the persistent catabolic burden and progressive muscle loss that frequently extend well beyond discharge [18, 86, 89]. Nutritional status is emphasised as a major determinant of functional recovery, yet the acute phase is characterised by inflammation-driven hypercatabolism that often cannot be reversed solely with nutritional therapy [9, 13]. An anabolic switch is required before patients can effectively rebuild FFM, positioning the rehabilitation phase as a critical window for targeted nutritional intervention. While the initial loss of muscle mass occurs rapidly, rebuilding it during rehabilitation requires long-term approaches. Efforts are already underway to ensure seamless care. The Continuum of Care concept refers to a patient-oriented workflow for modern nutrition management [90].

Findings from intensive care units, medical wards, and community facilities show varying treatment effects, which are inversely proportional to the disease severity [1, 91, 92]. The time window for nutritional therapy after discharge is promising. However, many studies were conducted only during hospitalisation and did not show a lasting positive impact on survival [93]. The ongoing EFFORT II trial (NCT04926597) aims to fill this gap by testing a structured, multidisciplinary continued outpatient nutrition program—combining dietitian home visits and telemedicine—designed to improve survival, reduce readmissions, enhance functional status, and support body composition recovery. With a target sample size of 802 to 1,200 participants and event-driven design, EFFORT II will provide critical insights into whether comprehensive transitional nutrition care can meaningfully affect long-term outcomes.

### Are guidelines still required?

While the ESPEN-ICU guidelines are under revision, many voices question their utility now that the personalised strategy is advocated. Guidelines do not preclude individual attention, but they ensure the basal SOP, including evaluation of the patients and monitoring the result of the therapy. The importance of education is recognised [94]. But despite significant efforts including by ESPEN and ASPEN, the education level in nutrition remains sadly low, and absence of local feeding protocols is widespread resulting in gaps between practice and basal recommendations [95]. Moreover although many ICUs have generated protocols, their application faces the absence of adequate monitoring resulting in highly variable energy and protein deliveries as shown in the EuroPN study [46] and studies based on NutritionDay [96, 97]. Therefore, despite their weakness, guidelines remain the basis enabling orienting nutrition therapy based on evidence.

## Conclusion

Critical illness induces profound and rapidly evolving metabolic disturbances that challenge the effectiveness of conventional nutritional strategies. Evidence presented in this symposium reinforces that individualisation, not uniformity, is central to effective nutrition therapy. Inflammation, catabolism, body composition, and metabolic tolerance vary widely between patients and over time, necessitating adaptive approaches guided by indirect calorimetry, fat-free mass estimates, and emerging biomarkers such as the urea-creatinine ratio.

Micronutrient deficiencies are common and clinically relevant, requiring systematic assessment and phase-appropriate supplementation and completion. Macronutrients should be viewed as pharmacologic agents, delivered progressively and titrated to metabolic capacity rather than static targets. Novel metabolic interventions, such as structured ketogenic diet protocols in certain patient populations like those with refractory neurological disease or sepsis, show promise when implemented within standardised, interdisciplinary frameworks.

Beyond the ICU, survivors remain at high risk for malnutrition, muscle loss, and functional decline. Integrating nutrition into multimodal rehabilitation, ensuring early recognition of PICS, and establishing structured transitional care pathways may help reverse the long-term consequences of critical illness. Ongoing trials, including comprehensive outpatient programs, are expected to clarify how sustained, individualised nutrition can influence long-term survival and recovery.

Overall, a precision-based, phase-specific, and interdisciplinary approach to nutrition therapy across the entire course of critical illness, from the acute phase through rehabilitation, holds substantial potential to improve outcomes and enhance quality of life for critically ill patients.

## Data Availability

No datasets were generated or analysed during the current study.

## Abbreviations

BHB: β-Hydroxybutyrate
CRP: C-reactive protein
DGEM: German society for nutritional medicine
DHA: Docosahexaenoic acid
ECMO: Extracorporeal membrane oxygenation
EPA: Eicosapentaenoic acid
ESPEN: European society for clinical nutrition and metabolism
FFM: Fat-free mass
FIRSES: Febrile infection-related epilepsy syndrome
GDF15: Growth/differentiation factor 15
ICU: Intensive care unit
IL-6: Interleukin-6
KD: Ketogenic diet
MCT: Medium-chain triglyceride
MN: Micronutrient
NORSE: New onset refractory status epilepticus
PICS: Post-Intensive Care Syndrome
PUFA: Polyunsaturated fatty acid
REE: Resting energy expenditure
RSE: Refractory status epilepticus
SOP: Standard Operating Procedure
SRSE: Super-refractory status epilepticus
UCR: Urea-creatinine ratio
